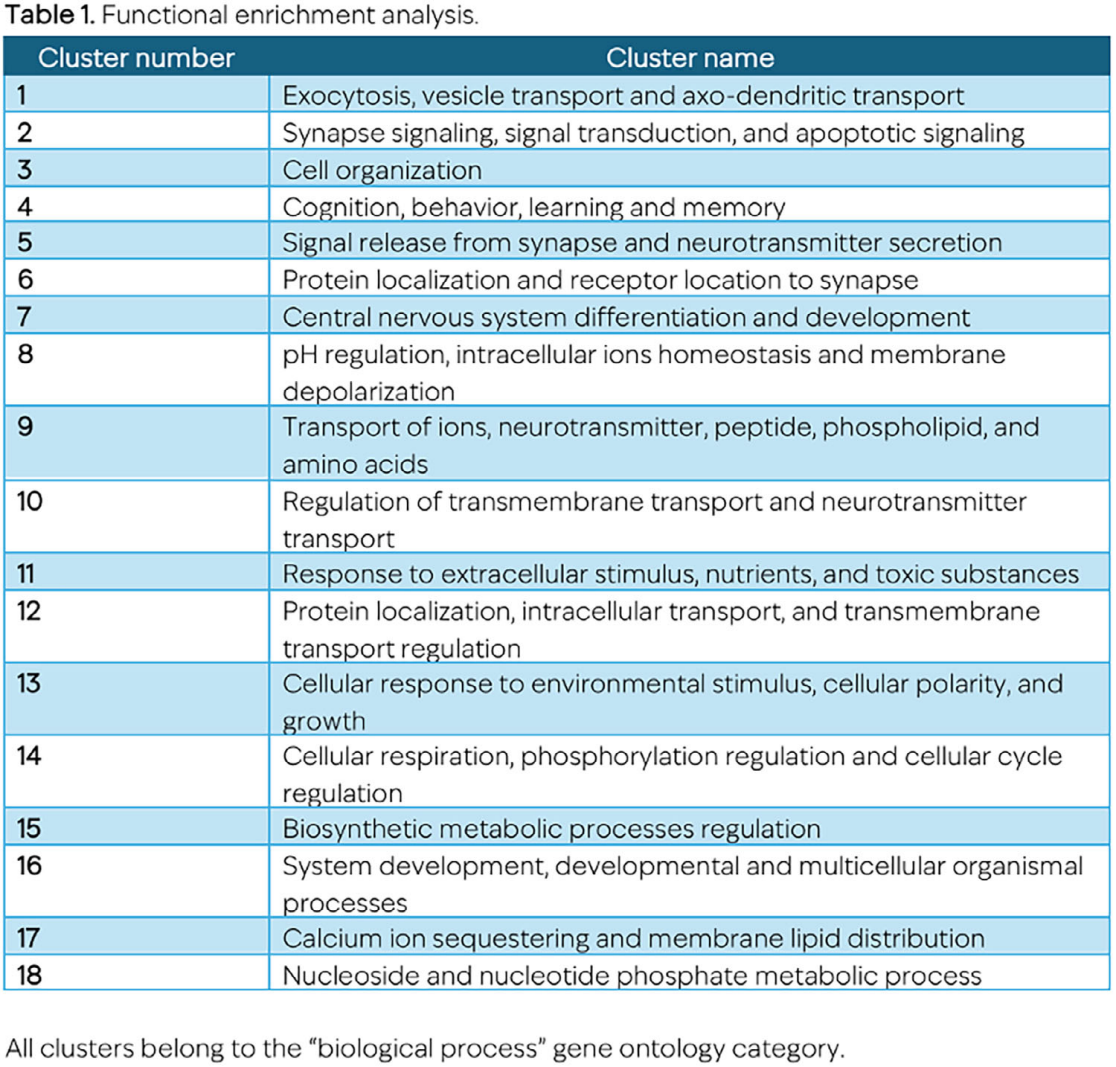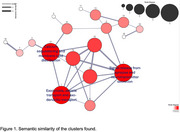# A Semantic Similarity Analysis in the brains of Alzheimer's disease and COVID‐19 deceased individuals

**DOI:** 10.1002/alz70861_108309

**Published:** 2025-12-23

**Authors:** Guilherme Bastos de Mello, Giovanna Carello‐Collar, Débora Guerini de Souza, Marco Antônio De Bastiani, Eduardo R. Zimmer

**Affiliations:** ^1^ Pontifícia Universidade Católica do Rio Grande do Sul, Porto Alegre, RS Brazil; ^2^ Universidade Federal do Rio Grande do Sul, Porto Alegre, RS Brazil; ^3^ Brain Institute of Rio Grande Do Sul, PUCRS, Porto Alegre, RS Brazil; ^4^ Universidade Federal do Rio Grande do Sul, Porto Alegre, Rio Grande do Sul Brazil; ^5^ McGill University, Montreal, QC Canada

## Abstract

**Background:**

Neurological manifestations, such as impaired memory, reduced attention, and cognitive decline, are frequently reported in individuals with COVID‐19 and resemble symptoms observed in Alzheimer’s disease (AD). Nonetheless, the extent to which these two conditions share molecular patterns remains uncertain. This study investigates transcriptomic parallels in the frontal cortex of patients with COVID‐19 and AD.

**Method:**

Transcriptomic datasets from the frontal cortex of COVID‐19 and AD patients were retrieved from the Gene Expression Omnibus. We used the R statistical software to identify differentially expressed genes (DEGs) in COVID‐19 and AD brains compared to controls (FDR‐adjusted *p* ‐value <0.05). Functional enrichment analysis (FEA) was applied to explore biological processes enriched in the shared DEGs.

**Result:**

A total of 6,533 DEGs were identified in COVID‐19 samples and 5,592 in AD samples. Among them, 2,789 DEGs were shared between both conditions. Functional clustering (Table 1) and semantic similarity of the overlapped genes revealed significant convergence in processes related to vesicle transport, exocytosis and regulated secretion (Figure 1). The predominant terms indicate active cytoskeleton‐dependent intracellular trafficking, particularly involving the release of neurotransmitters, hormones, and signaling molecules. Additionally, processes involving calcium ion sequestration and membrane lipid distribution suggest integrated mechanisms of signaling and cellular homeostasis.

**Conclusion:**

These findings point to a functional overlap between the pathophysiological mechanisms of Alzheimer's disease and the effects of COVID‐19 in the brain, highlighting vulnerabilities in intercellular communication and regulation of the cellular microenvironment, as an outcome of neuroinflammatory vulnerabilities. These findings could guide clinical approaches for monitoring and managing cognitive impairments in COVID‐19 survivors who may be at increased risk of neurodegenerative diseases like AD.